# “Stand by me”: astrocyte–microglia crosstalk in central nervous system development, homeostasis, injury, and disease

**DOI:** 10.3389/fnetp.2026.1895622

**Published:** 2026-09-15

**Authors:** Alberto Javier Ramos, Alejandro Villarreal

**Affiliations:** 1 Instituto de Biología Celular y Neurociencia “Prof. E. De Robertis” UBA-CONICET, Facultad de Medicina, Universidad de Buenos Aires, Buenos Aires, Argentina; 2 Instituto Tecnológico de Buenos Aires (ITBA), Buenos Aires, Argentina

**Keywords:** astrocytes, crosstalk, homeostasis, microglia, network physiology, neurodevelopment, pathology

## Abstract

During central nervous system (CNS) development, astrocytes and microglia arise from distinct embryonic origins; however, during early development, both cell types coordinately participate in brain circuit formation and neuronal network establishment. Later, in the healthy adult CNS, astrocytes are known to play essential roles in maintaining homeostasis. Although the microglia primarily function as the resident immune cells of the CNS, it is now well established that these cells, together with the astrocytes, participate actively in synaptic regulation, neuronal circuitry functioning, and cognition. In response to acute injury or chronic disease, both cell types undergo profound phenotypic changes and engage in a dynamic bidirectional crosstalk that critically shapes neuroinflammatory responses and influences CNS damage progression. This review synthesizes current knowledge and provides updated insights into the mechanisms by which astrocytes and microglia communicate during embryonic development of the CNS, participate in network physiology in the adult CNS, and engage in cellular responses to disease, highlighting how their interactions contribute to pathology and enabling identification of potential avenues for therapeutic interventions. Specifically, we focused on reviewing articles that directly address astrocyte–microglia communication. As an interesting outcome of our literature survey, we identified a significant bias toward studies investigating astrocyte–microglia crosstalk in pathological contexts compared to development and healthy adulthood. This observation reveals underexplored research avenues and emphasizes the need for studies addressing the roles of astrocyte–microglia interactions during physiological development and adult homeostasis. Finally, we briefly review novel approaches to overcome the difficulties of addressing astrocyte–microglia crosstalk.

## 1 Introduction

Astrocytes and microglia arise from distinct embryonic origins during the development of the central nervous system (CNS). Microglia are of myeloid origin and are the macrophages in the CNS as well as canonical immune cells. Astrocytes arise from neural precursor cells following microglial entry into the CNS. Once the CNS matures and cells reach their functional locations, both astrocytes and microglia participate in maintaining tissue homeostasis. Finally, both cell types participate actively in the cellular responses during aging, injury, neurodegenerative disease, and pathological states in general.

Both astrocytes and microglia have very important functions during CNS development and homeostasis; moreover, both types of cells are highly involved in pathology initiation and progression. The roles of both cell types in all these processes have been widely investigated; although their interactions are often considered, little is really known about their crosstalk mechanisms. In this review, we discuss the current knowledge on astrocyte–microglia crosstalk and highlight the difficulties of studying such interactions in terms of models and approaches.

## 2 Methods

To obtain an overview of the distribution of studies investigating astrocyte–microglia crosstalk across different biological contexts, a structured search was performed in PubMed using predefined combinations of keywords related to astrocytes, microglia, and intercellular communication. The searches were organized into mutually exclusive categories representing pathological conditions, development, and adult homeostasis/physiology. Additional exclusion criteria were applied to reduce overlap and remove non-primary literature, including review articles, editorials, commentaries, methodological articles, preprints, and studies focusing primarily on immortalized cell lines or intervention-driven paradigms. The search results were manually inspected to minimize misclassifications and improve contextual relevance. In addition, the reference lists of relevant articles were manually searched to identify further studies that met the inclusion criteria. This search strategy was intended to provide a contextual overview of the available literature rather than a formal systematic or bibliometric analysis (see Supplementary Material for the PubMed search strategies). Because the primary objective of this review was to evaluate experimental evidence for astrocyte–microglia crosstalk, only original research articles reporting primary data were considered when analyzing the crosstalk mechanisms. Nevertheless, review articles were used for exploring the background and summarizing established concepts in astrocyte and microglial biology to provide context and identify additional relevant primary studies. The structured search resulted in substantially different numbers of publications across the three biological contexts: 28 records for development, 94 records for homeostasis, and 1,554 records for pathology. No restrictions were applied on the publication year, and all records indexed in PubMed from database inception through 5 July 2026 were eligible for screening. The literature search was conducted between 1 May and 5 July 2026.

### 2.1 The breakfast club: different origins and the unlikely astrocyte–microglia interactions

#### 2.1.1 Origins and functions of microglia and astrocytes during CNS development

Microglia are early-arriving macrophages arising from the hematopoietic stem cells in the yolk sac as “primitive macrophages” that migrate into the developing brain around the 4^th^ gestational week in humans and prior to embryonic day 8 in mice, colonizing different CNS regions (Ginhoux et al., 2010; Menassa and Gomez-Nicola, 2018; Reemst et al., 2016; Rezaie and Male, 2002).

Astrocytes do not share an embryological origin with microglia. Like neurons and oligodendrocytes, astrocytes are of neuroectodermal origin. During brain development, the neural progenitors (radial glial cells) sequentially produce neurons, astrocytes, and oligodendrocytes (Götz and Huttner, 2005; Villarreal and Vogel, 2021; Vivi and Di Benedetto, 2024). In humans, astrogliogenesis occurs throughout the second half of fetal development with regional variations; in rodents, astrogliogenesis occurs around embryonic day 18; in both cases, astrogliogenesis occurs after the peak of neurogenesis (Holst et al., 2019; Miller and Gauthier, 2007; Reemst et al., 2016).

During CNS development, microglia have many functions, such as synapse pruning, thus collaborating in the formation of appropriate neuronal circuits: a phenomenon that depends on synapse decoration with “eat-me” signals, such as complement-derived peptides. Furthermore, the microglial cells can influence neuronal differentiation and angiogenesis (Geloso and D’Ambrosi, 2021; Reemst et al., 2016; Scott-Hewitt et al., 2020; Stevens et al., 2007). Altered pruning during critical periods of development is hypothesized to contribute to the development of human pathologies, such as schizophrenia and autism disorders (Beopoulos et al., 2022; Boksa, 2012; Howes and Onwordi, 2023).

#### 2.1.2 Evidence of “early” crosstalk

Astrocytes and microglial cells have been widely studied separately or in relation to neurons individually. For example, both cell types are involved in the process of neurogenesis (Araki et al., 2021); however, little is known about how each cell type influences the other (Reemst et al., 2016).

Using *in vitro* models with neural progenitor cells, it was shown that microglia-derived factors like interleukin 6 (IL-6) and leukemia inhibitor factor (LIF) influence astrocyte differentiation (Antony et al., 2011; Nakanishi et al., 2007). However, more research is required in this context. Although available data may not evidence early astrocyte–microglia crosstalk, it has been suggested that microglia-mediated phagocytosis of recently differentiated cells could regulate neuron (Cunningham et al., 2013) and astrocyte numbers (VanRyzin et al., 2019). Chung et al. (2013) showed evidence of astrocyte participation in synapse pruning during development through the multiple epidermal growth factor-like domains 10 (MEGF10) and MER tyrosine kinase (MERTK) pathways and recognition of “eat-me” signals. However, this function has been mainly attributed to microglia. Interestingly, even when microglia might be the main players in synapse engulfment, it has been reported that such mechanisms highly depend on astrocyte participation. Astrocytes can promote microglia functions in synapse engulfment by secreting IL-33 during spinal cord development (Vainchtein et al., 2018). During brain cortical postnatal development and upon whisker stimulation in mice, the microglia instruct astrocytes via wingless related integration site (Wnt) to disengage synapses prior to their engulfment by the microglia (Faust et al., 2025). During early postnatal thalamic development, astrocytes signal the microglia through Hevin–toll-like receptor 4 (TLR4) interactions, promoting TLR2 expression and phagolysosomal activity in the microglia along with synapse removal (Ramirez et al., 2026).

Summarizing the available evidence, we note that microglia might participate in astrocyte differentiation during CNS development, a phenomenon consistent with the fact that microglia temporarily precede astrocyte residence in the brain (Pont-Lezica et al., 2011; Reemst et al., 2016). Once the astrocytes mature and begin participating in synaptic functions, they also contribute to neuronal circuitry establishment by promoting microglia-dependent engulfment. Collectively, the available evidence suggests that astrocyte–microglia communication evolves throughout CNS development. Early in the development, microglia likely contribute to astrocyte differentiation and maturation, consistent with their earlier colonization of the CNS. As astrocytes mature, they progressively acquire the ability to regulate microglial functions involved in synaptic refinement and circuit formation. Although relatively few studies have directly addressed reciprocal signaling, the available data support the concept that bidirectional astrocyte–microglia communication is established during development and provides the foundation for glial interactions later in life.

### 2.2 Guardians of the galaxy: astrocytes and microglia as keepers of CNS homeostasis

#### 2.2.1 Astrocyte homeostatic functions and microglial surveillance

Once mature, astrocytes play important roles in maintaining brain homeostasis; such functions have been widely reviewed elsewhere and represent an important research focus since any alterations might affect neuronal performance and survival. In brief, astrocytes are metabolically coupled with neurons through mechanisms, such as the lactate shuttle (Barros, 2013; Barros et al., 2020; Barros and Weber, 2018). Astrocytes can participate actively in synaptic functions by physically wrapping around them, expressing proteins such as glutamate receptors and transporters like excitatory amino acid transporter 2 (EAAT2; also known as glutamate transporter 1 or GLT-1) and EAAT1 (also known as glutamate–aspartate transporter or GLAST), releasing gliotransmitters, and evoking intracellular calcium responses, among other functions (Araque et al., 1999; Escartin et al., 2021; Lines et al., 2020; Mahmoud et al., 2019; Navarrete et al., 2012; Perea et al., 2009; Shinozaki et al., 2014). Astrocytes couple neuronal functions with microvascular blood flow by sensing firing activity through mechanisms involving K^+^ buffering and by regulating vascular functions (Wallraff et al., 2006; Witthoft et al., 2013). Although astrocytes specifically sense synaptic activities in each of their domains (Benoit et al., 2025), they are largely coupled by gap junctions and can thus integrate across wide regions of the brain by forming a syncytium involving connexins (Cooper et al., 2026; Kiyoshi and Zhou, 2019). Finally, astrocytes are functional components of the blood–brain barrier (BBB) and regulators of water homeostasis in the CNS by expressing aquaporin 4 (AQP4) channels (Abbott et al., 2006; Daneman and Prat, 2015; Salman et al., 2022). Astrocytes are also implicated in myelin homeostasis, as reviewed elsewhere (Kıray et al., 2016).

Similarly, microglia contribute to and regulate synaptic functions (Badimon et al., 2020; Basilico et al., 2022; Clark et al., 2015; Liu et al., 2021; Schafer et al., 2013). Interestingly, it has been recently shown that microglia play relevant roles in maintaining myelin integrity after myelin ensheathment occurs during development (McNamara et al., 2023). As professional immune cells, the typical roles of microglia in homeostasis also involve homeostatic surveillance in the CNS (Arcuri et al., 2017; Nimmerjahn et al., 2005; Prinz et al., 2019; Salter and Stevens, 2017).

It has also been widely proposed that interference with astrocyte and microglial functions in the healthy brain could lead to loss of CNS homeostasis and likely pathological-like states (Collier et al., 2025; Cuautle et al., 2024; Gómez Cuautle et al., 2026; Rial et al., 2015). Owing to their functions in these homeostatic processes, specifically in synaptic and circuit functions, astrocytes and microglia have been implicated in behavior and memory (Adamsky and Goshen, 2018; Escalada et al., 2024; Ortinski et al., 2022; Wang et al., 2020). However, specific crosstalk was not addressed in such paradigms.

#### 2.2.2 Astrocyte–microglia crosstalk in homeostatic “healthy” CNS

The available literature on astrocyte and microglial functions in homeostasis is robust and has been evolving over the years. Mechanisms such as regulation of synaptic activity are shared and have led to the hypothesis that both cell types might be interacting in these and other processes. However, the manner in which astrocytes and microglia regulate each other under homeostatic conditions remains poorly understood. Several studies modified microglial states and reported impacts on astrocyte and synapse functions, but such works achieved microglial activation using proinflammatory stimuli (Clark et al., 2015; Rothhammer et al., 2018). Although this suggests astrocyte–microglia interactions, the stimuli used probably resembled a pathological situation.

Although certain mechanisms were not studied in the context of astrocyte–microglia communication, such crosstalk can be inferred. Recent works show an important role of transforming growth factor β (TGF-β) in the microglia during homeostasis (Bedolla et al., 2024; Ware et al., 2026). Microglia can release trophic and immunomodulatory mediators, including TGF-β, IL-10, and insulin-like growth factor 1 (IGF-1) (Prinz et al., 2019; Salter and Stevens, 2017), which might restrain astrocytic inflammatory responses (Bellini et al., 2011; Benveniste et al., 1995).

The phagocytic roles of microglia have been widely reported as canonical functions of macrophages. Astrocytes and microglia might coordinate their roles in phagocytosis during processes like cell corpse clearance in a MERTK-dependent pathway (Damisah et al., 2020). Interestingly, it was reported that astrocytes can remove microglial debris through phagocytosis. Such a phenomenon is important in microglial turnover *in vivo* in a complement component 4b (C4b)-mediated manner (Zhou et al., 2022a). Finally, microglial pharmacological depletion was found to cause astrocyte syncytium uncoupling through downregulation of connexins 30 and 43 (Cx30 and Cx43, respectively) in the mouse hippocampus (Du et al., 2022), suggesting microglial regulation of the astrocyte network and homeostatic functions.

Overall, the available evidence indicates that astrocyte–microglia crosstalk during physiological homeostasis remains considerably less understood than in pathology. Existing studies suggest that reciprocal signaling contributes to the maintenance of synaptic functions, phagocytic activities, and glial network organization. However, because many experimental approaches rely on inflammatory stimulation or cell depletion, distinguishing physiological communication from secondary responses remains a major challenge. Addressing this question will be essential for understanding how astrocytes and microglia cooperate to preserve CNS homeostasis.

### 2.3 Wild tales: when astrocyte–microglia crosstalk spirals out of control

#### 2.3.1 Microglia as mediators of astrocytic responses to inflammatory stimuli

Upon injury or disease, both astrocytes and microglia participate actively in the cellular response generically known as reactive gliosis. Depending on the origin of the injury, reactive gliosis has different outcomes, for example, promoting different degrees of neuroinflammation (Allahyari and Garcia, 2015; Burda and Sofroniew, 2014; Cieri and Ramos, 2025; Escartin et al., 2021; Pekny and Nilsson, 2005; Ransohoff, 2007; Sofroniew, 2020; 2015; Streit et al., 1999; Villarreal et al., 2016). Microglial responses and participation in neuroinflammatory responses have been documented and reviewed extensively (DeMeulenaere et al., 2026; Gao et al., 2023; Leng and Edison, 2020; Muzio et al., 2021; Prinz et al., 2021; Shao et al., 2022).

Although microglial cells are the immune cells in the CNS, it has been largely demonstrated that astrocytes also participate actively in the innate immune response in the CNS (Acaz-Fonseca et al., 2019; Escartin et al., 2021; Hasel et al., 2021; Hyvärinen et al., 2019; Lee et al., 2023; Liddelow and Barres, 2017; Mayo et al., 2014; Novakovic et al., 2023; Rama Rao and Kielian, 2015; Wheeler et al., 2019). Injury is known to induce astroglial expression of innate immunity receptors, such as TLR2, TLR4, receptor for advanced glycation end-products (RAGE), and scavenger receptors, allowing astrocytes to respond to both pathogen-associated molecular patterns (PAMPs) and damage-associated molecular patterns (DAMPs) leading to activation of transcription factor NF-κB (Cuautle et al., 2024; Gorina et al., 2011; Hsiao et al., 2013; Nakano et al., 2015; Rama Rao and Kielian, 2015; Rosciszewski et al., 2019; Rosciszewski et al., 2017; Villarreal et al., 2021; Villarreal et al., 2014). Activation of NF-κB in the astrocytes was linked to a reactive phenotype with a proinflammatory gain of function (Hein et al., 2026; Lian et al., 2016; Liddelow et al., 2017; Rosciszewski et al., 2019; Rosciszewski et al., 2017; Villarreal et al., 2021; Villarreal et al., 2016; Villarreal et al., 2014; Wheeler et al., 2020). Activation of signal transducer and transcription activator 3 (STAT3) was also linked to certain types of astrocyte reactive phenotypes, particularly to astrocytes at injury borders (Anderson et al., 2016; Ceyzériat et al., 2016; Herrmann et al., 2008; Nakano et al., 2015; Wanner et al., 2013).

Astrocyte interactions with other cell types (neurons, endothelial cells, immune cells, etc.) have been described in different models, particularly under pathological conditions (Domingues et al., 2016; Han et al., 2021; Linnerbauer et al., 2020; Sofroniew, 2020). Moreover, particular attention has been devoted to interactions between astrocytes and immune cells (Dong and Benveniste, 2001; Han et al., 2021; Sanmarco et al., 2021).

Evidence from the past two decades appear to suggest a major role of microglia in astrocyte reactive phenotype acquisition; furthermore, evidence indicates that this is a two-way communication between both cell types that likely enhances or propagates initial signals. An experimental model of neuroinflammation with lipopolysaccharide (LPS) showed that astrocytes acquire a reactive phenotype with a proinflammatory gain of function after engaging with microglia-secreted interleukins (Liddelow et al., 2017). Using *in vitro* stimulation of primary astrocyte cultures with low and high abundances of microglia, our group showed that NF-κB activation is enhanced in cultures with high microglial density (Rosciszewski et al., 2019). This phenomenon of NF-κB activation is associated with morphological changes to the microglia and astroglia alongside expression of proinflammatory genes such as C3 (Villarreal et al., 2021). NF-κB activation on astrocytes was also shown to be microglia-mediated through *in vivo* models of inflammation (Lee et al., 2024; Wheeler et al., 2020). The dependence between microglial-derived molecules and propagation of reactive astrogliosis was demonstrated *in silico* and *in vivo* in different reports (Auzmendi et al., 2020; Cieri et al., 2023). Interestingly, it was also reported that STAT3 activation in reactive astrocytes at injury borders depends on microglia (Zhou et al., 2022b).

#### 2.3.2 Astrocytes as mediators of microglial responses to inflammatory stimuli

In contrast to studies on development and homeostasis, many articles and reviews have addressed the topic of astrocyte–microglia crosstalk in terms of pathology (Domingues et al., 2016; Escartin et al., 2021; Linnerbauer et al., 2020; Matejuk and Ransohoff, 2020; Sofroniew, 2020; Sun et al., 2023; Yang et al., 2025). During neuroinflammatory and neurodegenerative conditions, the reciprocal communication between microglia and astrocytes is altered profoundly, establishing a self-amplifying cycle that exacerbates tissue damage. Microglia are typically the first responders to CNS injury and release proinflammatory mediators, including tumor necrosis factor α (TNF-α), IL-1α, and C1q, upon activation to synergistically induce transition of astrocytes toward inflammatory reactive states characterized by impaired homeostatic functions and secretion of neurotoxic molecules (Escartin et al., 2021; Liddelow et al., 2017). In turn, the reactive astrocytes amplify microglial activation by releasing cytokines like IL-6 and granulocyte and macrophage colony-stimulating factor (GM-CSF), chemokines like C-C motif chemokine ligand (CCL2) and gamma-interferon-inducible protein 10 (CXCL10), complement proteins, adenosine triphosphate (ATP), and DAMPs, thereby sustaining leukocyte recruitment and chronic neuroinflammation (Jha et al., 2019; Matejuk and Ransohoff, 2020). Crosstalk between these glial populations is further modulated by extracellular vesicles, purinergic signaling, inflammasome activation, and alterations in lipid metabolism, which together influence phagocytosis, synaptic pruning, BBB integrity, and neuronal survival (Escartin et al., 2021; Prinz et al., 2019). Recent single-cell transcriptomic studies have demonstrated that both astrocytes and microglia exhibit remarkable phenotypic heterogeneity in disease, hinting at multiple context-dependent reactive states rather than discrete activation phenotypes. Consequently, the pathological outcomes are determined by not only the activation status of each glial population but also the dynamic and reciprocal interactions between them, making the astrocyte–microglia axis an attractive therapeutic target for epilepsy, Alzheimer’s disease, Parkinson’s disease, multiple sclerosis, traumatic brain injury, and other neurological disorders (Escartin et al., 2021; Masuda et al., 2020; Paolicelli et al., 2022).

During CNS development, the microglia are guided to synapse or neuronal engulfment through “eat-me” signals that counterbalance “don’t eat me” receptors. The effectors of this phagocytic pathway include proteins from the complement cascade. Later in life, these mechanisms seem to re-emerge in response to pathology. The pathophysiological relevance of these pathways in astrocyte–microglia crosstalk has been demonstrated in neuropathic pain (Mou et al., 2022).

However, these complement-dependent pathways may also be generically involved in astrocyte–microglia crosstalk during reactive gliosis after acute injury and chronic disease, as explained below. This is an example of a development-related pathway of microglia–astroglial interactions that is recruited back during CNS injury. In brain stroke, the complement component 3a receptor (C3aR) pathway was implicated in reactive gliosis (Ahmad et al., 2020; Nagao et al., 2025; Pekna et al., 2023; Pekna et al., 2021; Pekna and Pekny, 2021; Stokowska et al., 2023); however, it is not yet clear whether this pathway mediates astrocyte–microglia communication. Although an increase in serum C3 was measured in human patients following ischemic stroke (Yang et al., 2021), suggesting a peripheral source of C3 upon disruption of the BBB, it was recently shown in a model of brain stroke that astrocytes might be a potential source of C3 (Zhang et al., 2026); interestingly, the authors also reported evidence that astrocytes from the injury border express C3, which is probably used to recruit C3aR-expressing microglia. In models of Alzheimer’s disease, the C3–C3aR pathway was found to be involved in fibrillar amyloid beta (Aβ) clearance by the microglia (Fu et al., 2012); however, later reports have revealed astrocytes as a potential source of C3 in a context in which microglia express C3aR (Lian et al., 2016; Litvinchuk et al., 2018; Wu et al., 2019). These works provide evidence for the involvement of the C3–C3aR pathway in astrocyte–microglia crosstalk in neurodegeneration. It was also shown in chronic pathologies and upon proinflammatory stimulation that the microglia signal to astrocytes through interleukins like IL-1a, C1q, and TNF, promoting astrocyte C3 expression and release that consequently activate the microglia and probably increase neuroinflammation over time (Liddelow et al., 2017). Such examples show that a proinflammatory gain of function in astrocytes is related to neurotoxicity.

Together, the above results suggest an early role of the C3–C3aR pathway during development and a later role in reactive gliosis, modulating astrocyte–microglia crosstalk. We hypothesize that this development mechanism is probably not the only one recruited by injured cells in the CNS and that further studies may address these parallel responses. Accumulating evidence suggests that many of the signaling pathways governing astrocyte–microglia communication during CNS development are reactivated following acute injury and in chronic neurological disorders. These evolutionarily conserved programs, which normally orchestrate microglial maturation, synaptic refinement, tissue remodeling, and the establishment of glial homeostasis, are repurposed in response to pathological stimuli. Prominent examples include the abovementioned complement cascade (C1q–C3–CR3/C3aR), which regulates developmental synaptic pruning but drives aberrant synapse elimination during neurodegeneration. In addition, IL-33–suppression of tumorigenicity 2 (ST2) promotes physiological microglia-mediated circuit refinement yet contributes to inflammatory remodeling after CNS injury; TGF-β signaling is essential for maintaining the homeostatic microglial phenotype, even though its disruption contributes to the loss of microglial homeostasis during chronic disease. Similarly, developmental pathways involving colony-stimulating factor 1 (CSF-1)/IL-34 and colony-stimulating factor 1 receptor (CSF1R), C-X3-C motif chemokine ligand 1 (CX3CL1) and C-X3-C motif chemokine receptor 1 (CX3CR1), purinergic signaling mediated by ATP and purinergic receptors P2Y12 and P2X7, and triggering receptor expressed on myeloid cells 2 (TREM2) and apolipoprotein E (ApoE) are redeployed to regulate glial activation, phagocytosis, synaptic remodeling, and immune responses in disease. Although these development-related pathways initially support tissue repair and restoration of homeostasis, their sustained activation promotes maladaptive astrocyte–microglia crosstalk, perpetuating neuroinflammation, synaptic dysfunction, and progressive neuronal damage (Butovsky et al., 2014; Escartin et al., 2021; Kana et al., 2019; Keren-Shaul et al., 2017; Krasemann et al., 2017; Liddelow et al., 2017; Prinz et al., 2019; Stevens et al., 2007; Vainchtein et al., 2018). Of note, we mentioned above that STAT3 is a transcription factor related to astrocyte reactivity; however, STAT3 is also involved in astrocyte differentiation during CNS development (Hong and Song, 2014; Villarreal and Vogel, 2021).

Together, these findings support the concept that pathological astrocyte–microglia crosstalk is not mediated by a single signaling pathway but rather by the coordinated reactivation of multiple developmental communication programs. Understanding how these pathways interact across different disease contexts will be critical for identifying therapeutic targets capable of modulating neuroinflammation while preserving physiological glial functions.

### 2.4 Edge of tomorrow: novel technologies to study astrocyte–microglia crosstalk

#### 2.4.1 Traditional approaches

In many studies, astrocytic and microglial responses to injury were studied as part of descriptive approaches to understand reactive astrogliosis. Although these kinds of studies can positively correlate the activation of both cell types, they are insufficient to establish causality. For instance, one can assess morphological changes to both cell types using histological techniques to demonstrate strong correlation.

In a second type of approach, one of the two cell types can be targeted using genetic or pharmacological tools while addressing cellular responses in both cell types. Genetic tools include, for example, transgenic animals or local viral induction with Aldehyde dehydrogenase 1 family member L1 (ALDH1L1) or GFAP-driven promoters to target astrocytic pathways (Escartin et al., 2021; Mudannayake et al., 2016). The report published by Tey et al. used a combination of genetic tools, including a confetti reporter under the microglia CX3CR1 promoter, which allowed the study of microglia clonal expansion (Tay et al., 2017). Together with novel genomic approaches and confocal microscopy, this type of transgenic animal allowed the characterization of different microglial subtypes (transcriptomically and epigenetically) in an animal model of Alzheimer’s disease (Ardura-Fabregat et al., 2025). Traditionally, cell-type-specific promoters can be combined with drugs, such as tamoxifen, for temporal control (Franz Villarreal et al., 2019). In addition, several pharmacological drugs that specifically target and deplete the microglia (Green et al., 2020) have been used in loss-of-function paradigms. These drugs rely on specific receptors expressed by the microglia. Some of these studies were explored above in models in which microglial cells were depleted. Although more complex owing to their stable cytoarchitecture and morphological interaction with neurons and oligodendrocytes, similar paradigms were explored to deplete astrocytes. The most common drug used to eliminate astrocytes in living tissue is L-alpha-aminoadipate (L-AAA), while fluorocitrate is frequently used to inhibit astrocyte metabolism temporarily (Davis et al., 2021; Kuter et al., 2019). Frequently, *in vivo* studies have been combined with *in vitro* approaches using different configurations of astrocytic and microglial cultures to draw conclusions in simpler systems (Badley et al., 2025; Rosciszewski et al., 2019; Villarreal et al., 2021; Wheeler et al., 2020). These are traditional 2D static cultures that can also be used to culture human cells (Santos et al., 2017). However, human *in vitro* approaches are discussed below.

#### 2.4.2 Novel approaches

In the past, new and interesting techniques were reported as promising approaches to address microglia–astrocyte interactions *in vivo and in vitro*, especially considering the heterogeneity of both cell types in response to injury and disease.

Starting with *in vitro* approaches, two conditions have been explored. First, the research groups started to shift toward 3D culture systems. One approach included the use of bioprinters and bioinks, which are already shown to be compatible with neural cells derived from human-induced pluripotent stem cells (Yan et al., 2024).

Another promising approach for generating human 3D structures is the use of organoids, which have been proven to reproduce certain features of human brain development (Glass et al., 2026). However, these organoids derived from the neural lineage include neither microglia nor vasculature. Recent articles have reported options to overcome this issue and shown that human organoids of neural origin, including human microglia, can be implanted into mouse brains to achieve vascularization (Mansour et al., 2018; Schafer et al., 2023).

The use of dynamic microfluidic cultures will also improve the way we study microglia and astrocytes. For example, the use of a microfluidic device in which human astrocytes and microglia were co-cultured to address communication under inflammatory conditions was reported recently (Tujula et al., 2025).

A different approach using microfluidics allows researchers to address microglial crosstalk at the level of one-to-one cellular communication (Wheeler et al., 2023). In brief, transgenic astrocytes carrying an NF-κB–EGFP reporter were stimulated and separated into single cells; in parallel, microglia were genetically edited with clustered regularly interspaced short palindromic repeats-associated protein 9 (CRISPR–Cas9) using a lentiviral library for genomic perturbations and separated into single cells. Next, one astrocyte and one microglia were placed into an oil droplet and allowed to communicate. After a certain amount of time, capsules containing NF-kB-activated astrocytes were sorted from non-activated capsules and guide RNAs were recovered, amplified, and sequenced. Thus, the technique named “systematic perturbation of encapsulated associated cells followed by sequencing” (SPEAC-seq) allows researchers to discover microglial mediators of astrocyte inflammatory activation (Wheeler et al., 2023).

The researchers who described the SPEAC-seq technique also reported a very interesting approach for addressing *in vivo* and *in situ* astrocyte–microglia interactions (Clark et al., 2021). In brief, the technique named “rabies barcode interaction detection followed by sequencing (RABID-seq) involves injecting a pseudotyped rabies virus designed to target astrocytes while carrying a barcoded mRNA. Inside the astrocyte, the virus replicates before infecting a neighboring cell, leaving a barcoded trace. Single-cell sequencing is then performed, and the astrocyte-interacting network (or connectome) is established by tracing the barcode using bioinformatics tools. RABID-seq was also used to address an immunosuppressive astrocyte subtype interacting with glioblastoma (Andersen et al., 2025).

The above approaches were developed very recently and used specifically to study astrocyte–microglia crosstalk. Although each technique has limitations and caveats, it represents a promising contribution to the field. Together, these emerging technologies complement traditional approaches by allowing studies of astrocyte–microglia interactions with increasing spatial, temporal, and molecular resolutions. Their integration with genetic and functional approaches is expected to substantially improve our understanding of glial communications in both physiological and pathological contexts.

## 3 Conclusions, limitations, and future directions

Individually, the roles of astrocytes and microglia in CNS development, homeostasis, and pathology have been studied extensively. Although much remains to be elucidated, it is now well established that both cell types participate actively in CNS physiology and are not merely stromal cells (see Figure 1 for a graphical summary and Table 1). In contrast, despite the widespread recognition that astrocytes and microglia communicate with each other, relatively few studies have directly addressed the mechanisms and functional consequences of this crosstalk in neurodevelopment and homeostasis.

**FIGURE 1:**
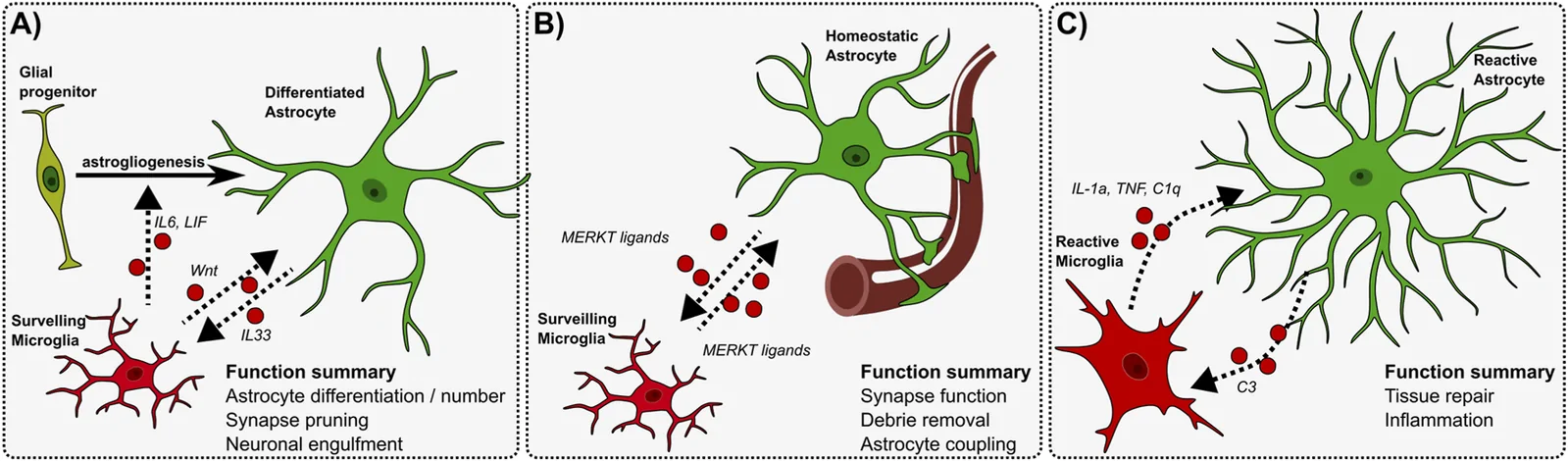
Astrocyte–microglia crosstalk across central nervous system (CNS) development, homeostasis, and pathological gliosis. Schematic representation of the bidirectional interactions between microglia (red) and astrocytes (green) during **(A)** development/early postnatal life, **(B)** homeostasis, and **(C)** gliosis. **(A)** During development, the surveilling microglia promote astrocyte differentiation (astrogliogenesis) and regulate astrocyte numbers by releasing soluble factors, including IL-6 and LIF. In turn, the astrocytes influence microglial functions through signals, such as Wnt and IL-33, thereby modulating synaptic pruning and neuronal engulfment. **(B)** During homeostasis, the astrocytes and surveilling microglia engage in reciprocal communication through MERTK ligands, contributing to synaptic functions, debris clearance, and maintenance of astrocyte coupling and CNS homeostasis. **(C)** Under pathological conditions, the reactive microglia secrete inflammatory mediators, including IL-1α, TNF, and C1q, which promote astrocyte reactivity. The reactive astrocytes subsequently release complement component C3 and other mediators that further modulate microglial activation, establishing a bidirectional inflammatory loop that contributes to tissue repair but may also sustain neuroinflammation. The dashed arrows indicate intercellular signaling mediated by representative soluble factors, whereas the functional summaries highlight the principal biological processes associated with astrocyte–microglia crosstalk at each stage.

**TABLE 1:** Summary of representative astrocyte–microglia communication pathways across CNS development, homeostasis, and pathology. The pathways are grouped according to biological context and classified as direct, functional, or correlative based on the level of experimental evidence supporting astrocyte–microglia communication.

Context	Communication pathway	Biological outcome	Evidence	Representative references
Development	IL-6/LIF	Astrogliogenesis	Direct	Ginhoux et al. (2013); Matejuk and Ransohoff. (2020)
	IL-33→ST2	Synaptic pruning	Direct	Vainchtein et al. (2018); Escartin et al. (2021)
	Wnt	Microglial maturation	Functional	Matejuk and Ransohoff. (2020)
Homeostasis	CSF-1/IL-34→CSF1R	Microglial survival	Direct	Butovsky et al. (2014); Salter and Stevens (2017)
	TGF-β	Homeostatic phenotype	Functional	Butovsky et al. (2014)
	ATP→P2Y12/P2X7	Surveillance	Functional	Salter and Stevens. (2017)
	CX3CL1→CX3CR1	Homeostatic communication	Correlative	Salter and Stevens. (2017)
Pathology	IL-1α/TNF/C1q	Reactive astrocytes	Direct	Liddelow et al. (2017)
	C3→C3aR	Neuroinflammation	Direct	Lian et al. (2016)
	IL-33→ST2	Tissue repair	Direct	Vainchtein et al. (2018)
	ATP→P2Y12/P2X7	Chemotaxis	Functional	Matejuk and Ransohoff (2020)
	Extracellular vesicles	Inflammatory signaling	Functional	Matejuk and Ransohoff. (2020)
	C1q–C3–CR3/C3aR	Synapse elimination	Functional	Escartin et al. (2021)

After reviewing current literature on astrocyte–microglia interactions, we draw the following conclusions:

1. During CNS development, the microglia influence astrocyte differentiation and likely their maturation, thereby contributing to regulation of astrocyte numbers.
2. During homeostasis, astrocyte–microglia communication remains poorly understood. Both astrocytes and microglia display substantial regional and functional heterogeneity throughout the CNS (Masuda et al., 2019; Schafer et al., 2023; Villarreal and Vogel, 2021), yet the extent to which reciprocal local interactions between these cell types contribute to the establishment or maintenance of this heterogeneity remains unknown.
3. Comparatively, a substantially larger number of studies have addressed astrocyte–microglia crosstalk under pathological conditions than during development or homeostasis. The importance of these interactions during disease is becoming increasingly evident, and many emerging experimental approaches summarized in our present review were originally developed to investigate astrocyte–microglia communication in the pathological context.
4. Cell depletion paradigms have been extensively used to investigate the functions of microglia and astrocytes to a lesser extent. However, the consequences of these approaches on normal CNS homeostasis have not been explored thoroughly. The extent to which eliminating one glial population alters the phenotype and functions of the remaining cell type remains unclear, potentially confounding the interpretation of crosstalk studies.

The current imbalance in literature likely reflects both technical and biological factors. During development and homeostasis, most experimental interventions—apart from transgenic approaches, non-invasive pharmacological manipulations, and descriptive analyses—can themselves activate the microglia and consequently the astrocytes, making it difficult to study physiological interactions without perturbing the system. This limitation has probably contributed to the relatively small number of studies addressing astrocyte–microglia communication under healthy conditions. In contrast, the clinical relevance of neuroinflammation has driven considerable interest in pathological settings, where astrocytes and microglia frequently operate as an interconnected inflammatory network. Thus, bidirectional communication becomes an explicit experimental target. However, during development and homeostasis, glial biology has historically been explored from a more cell-centric perspective, which may have obscured the contributions of physiological astrocyte–microglia interactions.

The evidence reviewed here indicates that astrocyte–microglia crosstalk begins early during CNS development and persists throughout adulthood and aging. Rather than acting as isolated responders, there is accumulating evidence that the astrocytes and microglia function as interconnected cellular partners whose molecular and functional interactions contribute to CNS development, homeostasis, and responses to injury. Under pathological conditions, physiological astrocyte–microglia communication may become maladaptive, generating self-amplifying inflammatory loops that contribute to chronic neurodegeneration. Emerging evidence further suggests that inflammatory signaling between these glial populations can promote epigenetic remodeling of the astrocytes, potentially contributing to altered metabolic support and other homeostatic functions. Collectively, the evidence reviewed here suggests that some glial responses traditionally interpreted as cell-autonomous may instead emerge from coordinated astrocyte–microglia interactions. Although this concept is well supported in several pathological settings, further studies are needed to determine its general applicability across developmental and homeostatic conditions. Future studies should therefore move beyond investigating astrocytes and microglia independently and instead focus on defining how their bidirectional interactions are regionally organized, temporally regulated, and dynamically modified across physiological and pathological states. A better understanding of these principles will be essential for deciphering CNS biology and for developing therapeutic strategies to modulate neuroinflammation without compromising the homeostatic functions of glial networks.

## Glossary

ALDH1L1: Aldehyde dehydrogenase 1 family member L1
ApoE: Apolipoprotein E
AQP4: Aquaporin 4
ATP: Adenosine triphosphate
Aβ: Amyloid beta
BBB: Blood–brain barrier
C1q: Complement component 1q
C3: Complement component 3
C3aR: Complement component 3a receptor
C4b: Complement component 4b
CCL2: C-C motif chemokine ligand
CNS: Central nervous system
CRISPR-Cas9: Clustered regularly interspaced short palindromic repeats-associated protein 9
CSF-1: Colony-stimulating factor 1 (macrophage colony-stimulating factor; M-CSF)
CSF1R: Colony-stimulating factor 1 receptor
Cx30: Connexin 30
CX3CL1: C-X3-C motif chemokine ligand 1 (fractalkine)
CX3CR1: C-X3-C motif chemokine receptor 1
Cx43: Connexin 43
CXCL10: Gamma-interferon-inducible protein 10
DAMP: Damage-associated molecular pattern
EAAT1: Excitatory amino acid transporter 1
EAAT2: Excitatory amino acid transporter 2
EGFP: Enhanced green fluorescent protein
GFAP: Glial fibrillary acidic protein
GLAST: Glutamate-aspartate transporter
GLT-1: Glutamate transporter 1
GM-CSF: Granulocyte and macrophage colony-stimulating factor
HEVIN: High endothelial venule protein
IBA1: Ionized calcium-binding adapter molecule 1
IL-1a: Interleukin-1 alpha
IL-33: interleukin 33
IL-34: Interleukin 34
IL-6: Interleukin 6
LIF: Leukemia inhibitory factor
LPS: Lipopolysaccharide
MERTK: MER tyrosine kinase
MEGF10: Multiple EGF-like domains protein 10
**NF-**κ**B**: Nuclear factor kappa B
P2X7: Purinergic receptor P2X7
P2Y12: Purinergic receptor P2Y12
PAMP: Pathogen-associated molecular pattern
RABID-seq: Rabies barcode interaction detection followed by sequencing
RAGE: Receptor for advanced glycation end-products
SPEAC-seq: Systematic perturbation of encapsulated associated cells followed by sequencing
ST2: Suppression of tumorigenicity 2
STAT3: Signal transducer and transcription activator 3
TGF-β: Transforming growth factor β
TLR2: Toll-like receptor 2
TLR4: Toll-like receptor 4
TNF: Tumor necrosis factor
TREM2: Triggering receptor expressed on myeloid cells 2
Wnt: Wingless-related integration site
