# Supplementary material for: “Stand by me”: astrocyte–microglia crosstalk in central nervous system development, homeostasis, injury, and disease
Source: Front Netw Physiol. 2026 Sep 15;6:1895622. doi: 10.3389/fnetp.2026.1895622 (PMC13628296; doi:10.3389/fnetp.2026.1895622)
Supplement: Supplementary file 1 [file DataSheet1.pdf]

Pathology (1063 hits)
(("astrocyte"[Title/Abstract] OR astrocytes[Title/Abstract])
AND("microglia"[Title/Abstract] OR microglial[Title/Abstract])
AND(crosstalk[Title/Abstract] OR "cross-talk"[Title/Abstract] OR communication[Title/Abstract] OR interaction[Title/Abstract] OR signaling[Title/Abstract]))
AND(disease OR pathology OR injury OR stroke OR trauma OR inflammation OR neurodegeneration OR tumor OR glioblastoma OR cancer)
NOT (development OR developmental OR maturation OR synaptogenesis OR homeostasis OR homeostatic OR physiological OR healthy)
NOT (protocol OR protocols OR method OR methods OR methodology OR preparation OR procedures OR experimental procedure
OR technical note OR assay development OR protocol development OR optimization)
NOT(Methods[Journal] OR "Methods Mol Biol")
NOT(Preprint[Publication Type] OR preprint[Title] OR bioRxiv OR medRxiv OR Research Square)
NOT(Comment[Publication Type] OR Editorial[Publication Type] OR News[Publication Type] OR Introductory Journal Article[Publication Type]
OR "News and Views" OR Preview OR Commentary OR "In this issue")
AND Journal Article[Publication Type]
NOT(Review[Publication Type] OR Systematic Review[Publication Type] OR Editorial[Publication Type] OR Letter[Publication Type] OR Preprint[Publication Type])

Development (28 hits)

(("astrocyte"[Title/Abstract] OR astrocytes[Title/Abstract])
AND ("microglia"[Title/Abstract] OR microglial[Title/Abstract])
AND (crosstalk[Title/Abstract] OR "cross-talk"[Title/Abstract] OR communication[Title/Abstract] OR interaction[Title/Abstract] OR signaling[Title/Abstract]))
AND (development OR developmental OR maturation OR synaptogenesis OR circuit formation OR brain development)
NOT (disease OR pathology OR injury OR stroke OR trauma OR inflammation OR neurodegeneration OR sepsis OR autism OR epilepsy OR epileptogenesis
OR seizure OR "febrile seizure" OR neurodevelopmental disorder OR maternal immune activation OR hypoxia OR ischemia OR glioblastoma OR tumor
OR cancer OR homeostasis OR homeostatic OR physiological OR healthy OR neonatal injury OR perinatal injury OR infection)
NOT (ethanol OR alcohol OR antipsychotic OR antipsychotics OR cannabinoid OR cannabinoids OR JWH-018 OR drug exposure
OR pharmacological treatment OR psychotropic OR substance abuse OR addiction)
NOT (forced exposure OR repeated exposure OR induced model OR chemically induced OR pharmacological induction)
NOT (protocol OR protocols OR method OR methods OR methodology OR preparation OR procedures OR experimental procedure OR technical note
OR assay development OR protocol development OR optimization)
NOT (Methods[Journal] OR "Methods Mol Biol")
NOT (Preprint[Publication Type] OR preprint[Title] OR bioRxiv OR medRxiv OR Research Square)
NOT (Comment[Publication Type] OR Editorial[Publication Type] OR News[Publication Type]
OR Introductory Journal Article[Publication Type] OR "News and Views" OR Preview OR Commentary
OR "In this issue") AND Journal Article[Publication Type] NOT (
Review[Publication Type] OR Systematic Review[Publication Type] OR Editorial[Publication Type] OR Letter[Publication Type]
OR Preprint[Publication Type])

Homeostasis (94 hits without reviews)
(("astrocyte"[Title/Abstract] OR astrocytes[Title/Abstract]) AND ("microglia"[Title/Abstract] OR microglial[Title/Abstract])
AND (crosstalk[Title/Abstract] OR "cross-talk"[Title/Abstract] OR communication[Title/Abstract] OR interaction[Title/Abstract]

OR signaling[Title/Abstract] OR regulation[Title/Abstract] OR modulation[Title/Abstract] OR maintenance[Title/Abstract]
OR phagocytosis[Title/Abstract] OR clearance[Title/Abstract] OR engulfment[Title/Abstract] OR pruning[Title/Abstract]
OR network[Title/Abstract] OR debris[Title/Abstract]))
AND (homeostasis OR homeostatic OR physiological OR healthy brain OR healthy adult
OR basal state OR normal function OR circuit refinement OR maintenance)
NOT (disease OR pathology OR injury OR stroke OR trauma OR inflammation OR neurodegeneration OR sepsis
OR autism OR epilepsy OR epileptogenesis OR seizure OR "febrile seizure" OR neurodevelopmental disorder OR maternal immune activation OR hypoxia
OR ischemia OR glioblastoma OR tumor OR cancer OR neonatal injury OR perinatal injury OR infection)

NOT(ethanol OR alcohol OR antipsychotic OR antipsychotics OR cannabinoid OR cannabinoids

OR JWH-018 OR drug exposure OR pharmacological treatment OR psychotropic OR substance abuse OR addiction)
NOT(forced exposure OR repeated exposure OR induced model OR chemically induced OR pharmacological induction)
NOT(protocol OR protocols OR method OR methods OR methodology OR preparation OR procedures OR experimental procedure
OR technical note OR assay development OR protocol development OR optimization)
NOT(Methods[Journal] OR "Methods Mol Biol") NOT(Preprint[Publication Type] OR preprint[Title] OR bioRxiv OR medRxiv OR Research Square)
NOT(Comment[Publication Type] OR Editorial[Publication Type] OR News[Publication Type] OR Introductory Journal Article[Publication Type] OR "News and Views"
OR Preview OR Commentary OR "In this issue") AND Journal Article[Publication Type] NOT (Review[Publication Type] OR Systematic Review[Publication Type]
OR Editorial[Publication Type] OR Letter[Publication Type] OR Preprint[Publication Type])
